# Spatial remapping improves reading with simulated central field loss

**DOI:** 10.1167/jov.26.4.11

**Published:** 2026-04-17

**Authors:** Colin S. Flowers, Arda Fidanci, Gordon E. Legge, Stephen A. Engel

**Affiliations:** 1 Department of Psychology, University of Minnesota, Minneapolis, MN, USA; 2 Center for Applied and Translational Sensory Sciences, University of Minnesota, Minneapolis, MN, USA

**Keywords:** low vision, reading, remapping, eye tracking, central field loss

## Abstract

One way to potentially aid reading with central field loss (CFL) is to spatially remap text outside the scotoma to appear in functioning visual field locations. We tested spatial remapping using a sentence reading task with typically sighted readers and simulated scotomata. Participants read sentences with six different remapping strategies of shifting text around the scotoma and with simulated scotomata of three shapes to assess (a) whether spatial remapping improved reading speed generally and (b) whether customization of spatial remapping based on the shape of the simulated scotoma provided additional improvement. Faster reading was observed in at least one remapping condition compared to a no-remapping condition with a simulated scotoma in all participants, with an average improvement of 23.80%. Customization was also important; the remapping strategies that resulted in the fastest reading depended on the shape of the scotoma. The results showed a strong effect of remapping on reading speed. Thus, customizable spatial remapping can aid reading in the presence of a simulated scotoma and should be considered for evaluation in patients with CFL, where it can be used to shift text to areas with functioning visual capabilities.

## Introduction

Reading greatly benefits from the high visual acuity afforded by central vision. However, there are situations when reading with central vision is not possible and understanding peripheral reading abilities and strategies is important. In particular, central field loss (CFL) arising from macular diseases obstructs reading and lowers quality of life (Hassell, Lamoureux, & Keeffe, 2006; Murro et al., 2017). During reading, the damaged area with affected visual capabilities, the *scotoma*, often occludes portions of text. A number of compensatory strategies have been investigated for reading in the presence of a scotoma, including magnification of text (Cheong, Lovie-Kitchin, Bowers, & Brown, 2005; Gill, Mao, Powell, & Sheidow, 2013), training of retinal fixation location (Nilsson, Frennesson, & Nilsson, 2003), perceptual learning to improve reading in peripheral vision (Chung, 2011), and training of eye movements (Nguyen, Stockum, Hahn, & Trauzettel-Klosinski, 2011; Seiple, Grant, & Szlyk, 2011; Seiple, Szlyk, McMahon, Pulido, & Fishman, 2005). In the current study, we evaluate another approach, with typically sighted individuals using peripheral vision to read in the presence of simulated scotomata: Text is *spatially remapped* such that letters never appear in the area that is occluded by a scotoma (Aguilar & Castet, 2017; Gupta et al., 2018; Ho, Loshin, Barton, & Juday, 1995; Loshin & Juday, 1989; Massof, Rickman, & Lalle, 1994; Wensveen, Bedell, & Loshin, 1995). We assess whether spatial remapping improves reading with eye movements and compare different strategies for remapping, along with the effects of different scotoma shapes.

**Figure 1.**
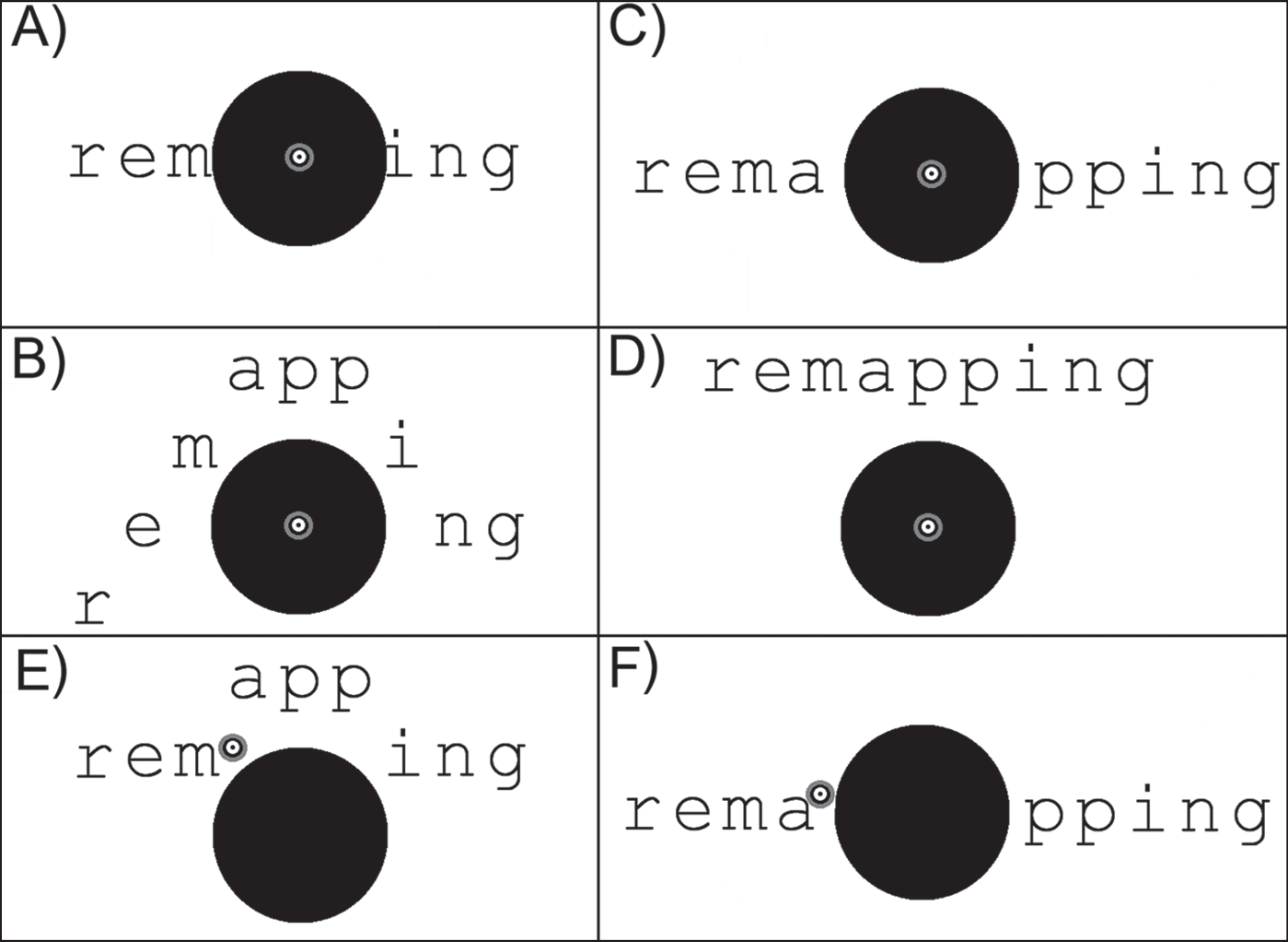
Examples of different spatial remapping strategies. The retinal location used for fixation is shown by the black and white annuli and corresponds to the anatomic fovea in typically sighted participants with a simulated scotoma: (**A**) “No remapping” results in letters being occluded by a scotoma, (**B**) “Diagonals” strategy curves around the top of a scotoma, (**C**) “Horizontal gap” strategy shifts letters to the left and right of the scotoma, and (**D**) “Max row” strategy shifts all letters above the scotoma in a horizontal row. Panels E and F show how spatial remapping can be extended to patients with CFL who have a scotoma: (**E**) “Vertical Gap” and (**F**) “Horizontal Gap.” In these final two panels, the black and white annuli correspond to a PRL that is adopted for fixation as the anatomic fovea is no longer functional. In CFL, spatial remapping would capitalize by placing letters at and near the PRL while avoiding the scotoma.

During remapping, as a person moves their gaze across the screen, text is shifted in real time in concert with eye movements so that letters are always presented on the functioning retina, bypassing the scotoma. In shifting the text, however, there are many different possible locations for each letter. For example, letters might be shifted to form diagonal contours that “hug” the border of the scotoma; they could be shifted horizontally, skipping over the scotoma; or they could be shifted as a whole straight row to be beneath or above the scotoma (see Figure 1). We refer to these heuristics for selecting letter locations as *strategies* and name these three as “diagonals,” “horizontal gap,” and “max row”—other remapping strategies are described below.

In the current study, we examine the impact on reading of the interaction of different-shaped scotomata and different remapping strategies. We use simulated scotomata in this study in typically sighted individuals who generally fixate words with their fovea (Figures 1A–D). Nevertheless, the same remapping strategies can be implemented in patients with CFL, who fixate with their preferred retinal locus (PRL), as the remapping strategies are designed to shift letters away from regions of poor letter perception, as illustrated in Figure 1E, F (for details of how remapping strategies are generated using letter-based perimetry maps, see Flowers, Legge, & Engel, 2024).

The scotoma shapes we use in this study do not exactly match particular scotomata in specific individuals with CFL, which vary greatly in size, shape, and continuity. Rather, a sample of different shapes that resemble general shape classes (“circular,” “wide,” “tall”), potentially resembling scotomata in patients, was selected. We did this so that the remapping strategies could be tested in a variety of different conditions (see customization hypothesis below). Nevertheless, the use of these shapes should be informative regarding how reading could be affected by remapping with scotomata that exhibit characteristics similar to the tested shapes. For example, in predicting a useful remapping for a scotoma that is wider than it is tall, the remapped reading results using the horizontally elongated simulated scotoma in the current study may serve as a useful starting point.

It may be that one strategy always results in the best reading, regardless of the shape of the scotoma. Alternatively, it may be that the best strategy will depend on the size or shape of the scotoma—a hypothesis that we refer to as the *customization hypothesis*. To parse these possibilities, we test with simulated scotomata of different shapes.

The remapping strategies were previously examined using a word recognition task with short presentations of individual words and assessing whether words could be seen in a single fixation (Flowers et al., 2024). However, eye movements are a critical component for typical reading (Rayner, 1978; Rayner, 1998; Rayner, Smith, Malcolm, & Henderson, 2009) and can be affected by CFL (Bullimore & Bailey, 1995; Rubin & Feely, 2009; Verghese, Vullings, & Shanidze, 2021; Wang, Zeng, Zhang, Mondal, & Zhao, 2023). It is reasonable to expect that gaze-contingent spatial remapping may also affect oculomotor control. In the current study, we measured sentence reading and eye movements to test whether the benefits of customized spatial remapping extend to sentence reading with eye movements. We hypothesized the following:

1. Spatial remapping would improve reading speed in the presence of a simulated scotoma.
2. Reading speed would vary as a function of both spatial remapping strategy and the shape of the simulated scotoma. That is, customized remapping strategies should produce the fastest reading.

## Methods

### Participants

Forty-one University of Minnesota (UMN) undergraduates participated for course credit or monetary compensation. All reported normal or corrected-to-normal vision and English as a native language. Technical difficulties prevented two participants from completing the experiment, and their data were excluded. Procedures were approved by the UMN Institutional Review Board and were in accordance with the Declaration of Helsinki.

### Apparatus

Stimuli were presented on an ASUS 24-inch LCD monitor running at 144 Hz with a 1,920-pixel × 1,080-pixel resolution. Participants viewed the screen from 60 cm (47.93° × 27.94°) with their head stabilized using a chinrest. Participants wore corrective lenses if prescribed to help them view the screen at that distance. The experiment was programmed in MATLAB (The MathWorks, Natick, MA, USA) with the Psychophysics Toolbox (Brainard, 1997; Kleiner et al., 2007) and Tobii SDK extensions. A second monitor displayed a live feed of the experiment viewed by the experimenter. A Tobii Spectrum Pro running at 300 Hz and down-sampled to 144 Hz monitored eye gaze, allowing eye-tracking data to be collected after every screen refresh.

### Stimuli

#### Characters

Letters (courier new font; x-width = 0.81°; letter spacing = 1.16x) were presented in black (0.39 cd/m^2^) on a white background (236 cd/m^2^).

#### Sentences

Sentences in the MNREAD test format (Calabrèse, Owsley, McGwin, & Legge, 2016; Calabrèse et al., 2018; Mansfield, Ahn, Legge, & Luebker, 1993; Mansfield, Atilgan, Lewis, & Legge, 2019) were randomly assigned to each trial and were not repeated. Sentences containing 9 to 15 words (*M* = 12.17, *SD* = 1.18) were broken into three segments corresponding to the three lines in MNREAD sentences. Each segment contained 2 to 6 words (*M* = 4.06, *SD* = 0.70) and 16 to 23 characters (*M* = 19.35, *SD* = 1.00). Each of the three segments was presented on a different display, one after another.

#### Dynamic gaze-contingent screen updating

**Figure 2.**
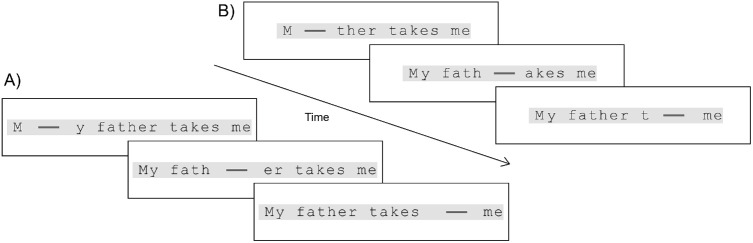
An example of three successive eye fixations while reading a display containing the first third of a sentence. The dark gray bar follows the center of gaze. (**A**) Horizontal gap remapping with a horizontally elongated simulated scotoma. (**B**) No remapping with a horizontally elongated simulated scotoma.

The displays that contained portions of sentences were updated each screen refresh based on the current gaze location. All simulated scotomata and remapped text were defined using an invisible grid (each location measured 1.1° × 1.7°). The nearest letter location to the current gaze position was selected to center the simulated scotoma and/or remapped text. To simulate a scotoma, letters were removed from the central corresponding grid locations, leaving only the gray background. A darker gray bar (58.3 cd/m^2^; see Figure 2) occupied the current gaze position and adjacent letter locations to the left and right, to help participants anchor their fixation and alert them if the eye tracker became miscalibrated.

Text was presented within a gray box (1.73° height; width varied with sentence length; 205 cd/m^2^), which designated where participants were instructed to look (see Figure 2). The gray box occupied the central row, and letters were always placed within the gray box for remapping conditions without any vertical displacement. Text was not presented if participants’ gaze left the gray box, even though remapped letters could be in these peripheral locations. The purpose of the gray box was to anchor the sentence in the middle of the screen and prevent participants from “chasing” remapped letters off-screen—a problem that emerged in piloting the task. The display was also blanked during a fast saccade (if the previous gaze location was more than 1.61° away, corresponding to a velocity of 232°/second). This was done to prevent participants from peeking “behind” the simulated scotoma.

#### Scotoma shapes

**Figure 3.**
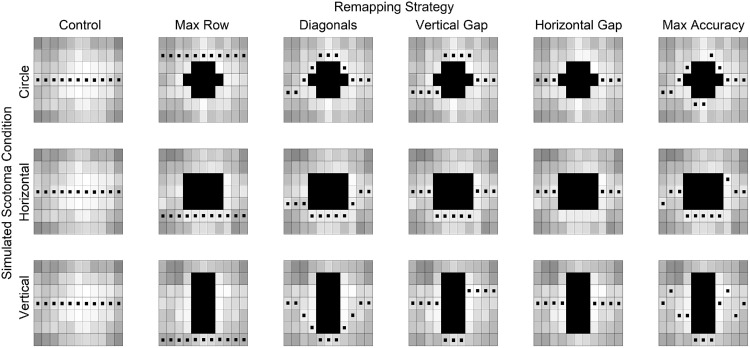
Remapping strategies tested. Columns index the different remapping strategies, and rows index the different simulated scotomata. In an additional no-remapping condition, letters are occluded by the simulated scotoma rather than remapped outside the scotoma. The solid black shapes in the center of each remapping show the simulated scotoma. The grayscale contrast of each location corresponds to letter recognition accuracy at that location from (Flowers et al., 2024), ranging from white (100%) to black (0%).

Three different simulated scotoma shapes were tested (see Figure 3; Flowers et al., 2024): (a) “circular,” (b) horizontally elongated, and (c) vertically elongated. The scotomata were constrained by the invisible grid used for letter placement, and this resulted in the scotomata not being precisely circular or oval. The circle and horizontally elongated scotomata measured 5.6° × 5.2°, the vertically elongated scotoma measured 3.4° × 8.6°, and they were roughly matched in area. Each participant viewed one of the three shapes.

#### Remapping strategies

The remapping strategies tested here were previously examined in a word recognition task (Flowers et al., 2024). The strategies were created by placing remapped text in areas of good letter recognition while following spatial patterns described below. Letter recognition was first assessed in a group of typically sighted participants across the locations of the invisible grid and served as the input to create the remapping strategies (Flowers et al., 2024). The invisible grid occupied a width of only 11 letter locations. Any letters that fell outside of this window were placed horizontally level with the letter located in the first or last column of the grid. The creation of these remapping strategies is flexible. Given a grid of letter recognition scores, remapping strategies can be computed following the spatial features outlined below. Here, these follow the sharply bounded simulated scotomata, but they need not. As they are computed using letter recognition maximization, they can also be created for more “noisy” grids that may be observed with CFL.

Six remapping strategies were tested along with a control condition as a within-participants factor; the control condition and the first five remapping strategies were also tested in Flowers et al. (2024; see Figure 3), with each being defined by its spatial features. (a) *Control* represented typical reading without any simulated scotoma or text shifting; (b) *Max Row*, where the entire line of text shifted above or below the simulated scotoma; (c) *Diagonals*, where letters were shifted to create diagonal patterns to avoid the simulated scotoma; (d) *Vertical Gap*, where a subset of the text shifted above or below to avoid the simulated scotoma; (e) *Horizontal Gap*, where letters were presented along the horizontal midline but shifted to the left and right so that all letters were still visible; (f) *Max Accuracy*, where letters in each column were presented in the row that maximized letter recognition accuracy (in the sample of participants from Flowers et al., 2024); and (g) *No Remapping*, where letters were presented in the same way as the control condition, but a simulated scotoma occluded central letters. The exact paths that these strategies followed (e.g., above or below the scotoma) depended on the observed letter recognition—paths that maximized letter recognition.

#### Eye tracker calibration

The eye tracker was calibrated at the start of each trial. Seven fixation dots (diameter = 0.25°) were presented sequentially, starting at the four corners and the center of the screen. The sixth dot was on the right side of the screen in the vertical center, and the final dot was on the left side of the screen in the vertical center, so that participants’ gaze was near the beginning of the sentence when it appeared. Participants were told the importance of eye-tracking calibration throughout the experiment and asked to fixate on each dot. They were asked not to anticipate the next fixation dot and wait until the current fixation dot disappeared before looking for the next one.

### Procedure

Participants read instructions on the screen and saw some sample pictures of stimuli to understand the task. There were opportunities for questions prior to practice and experimental trials. After experimental trials, participants were given a brief overview of CFL and were debriefed about the potential utility of text remapping. The entire study lasted under 2 hours.

#### Trial structure

**Figure 4.**
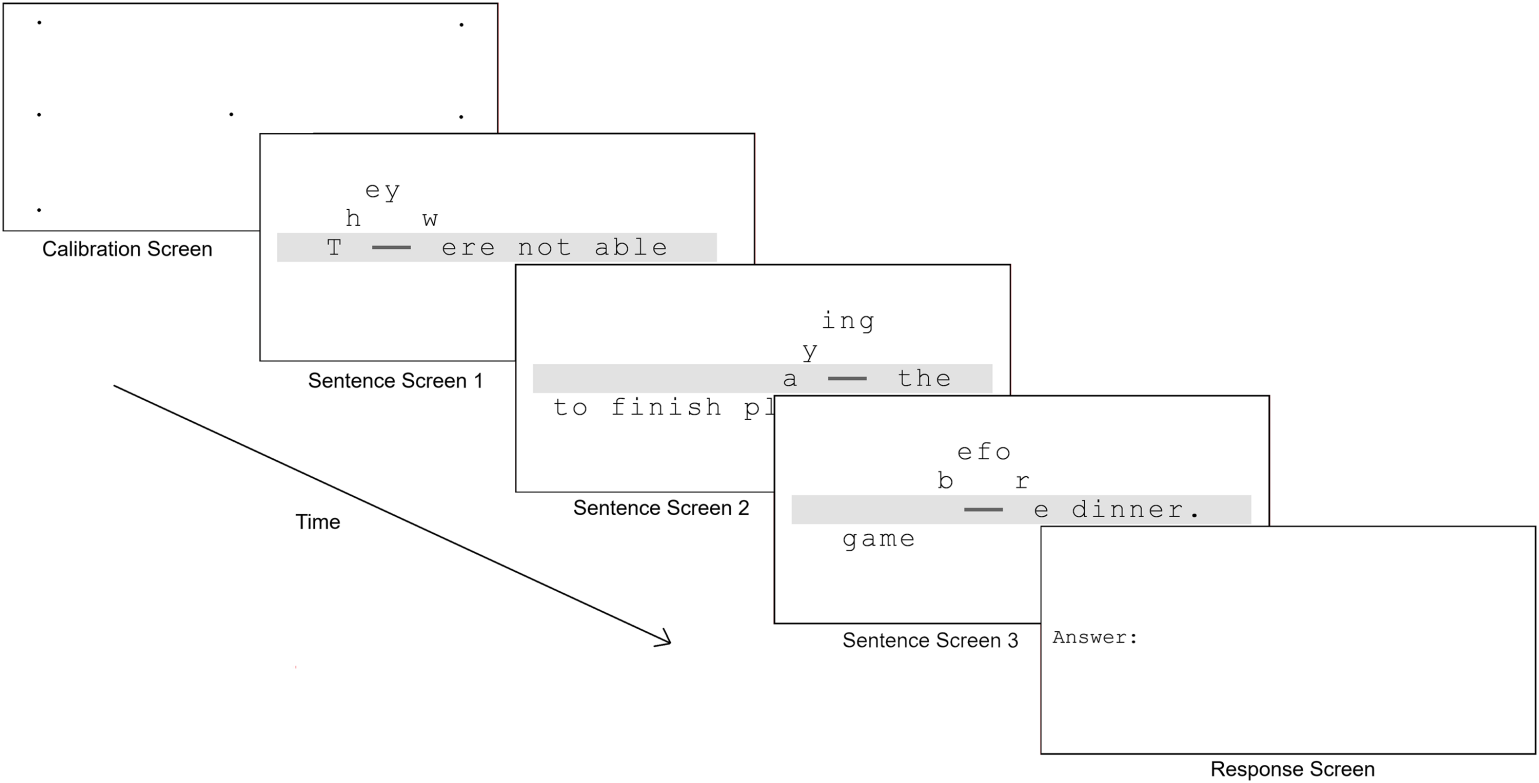
A sample trial with a circular scotoma and the diagonal remapping. The fixation dots on the calibration screen were presented sequentially. The three sentence displays continuously updated based on the current eye gaze on each screen refresh while being viewed. Participants pressed the space bar after reading the words on the screen to progress through the sentence.

Each trial (see Figure 4) began with the 7-point calibration followed by the sentence. Participants were asked to read aloud as they read a sentence and to press the spacebar when they finished the words on the screen containing the sentence. When they pressed the spacebar, the next screen with the next portion of the sentence was presented. After participants pressed the spacebar after reading the third and final screen, the experimenter recorded the number of errors—words omitted, replaced, or erroneously added. The experimenter also recorded any trials where there were issues with the eye tracker. This occurred (infrequently, 1.81% of all trials) due to poor calibration or head movements, and these trials were excluded from analyses. Participants were allowed to take breaks between trials as needed.

#### Practice trials

Participants initially completed 21 practice trials. Blocks of three trials for each remapping were completed in a random order. Each block started with a cue to preview where the letters would be presented relative to fixation, showing the remapping strategy. On the first few practice trials, participants were introduced to the task and encouraged to try moving their eyes back and forth across the gray box to familiarize themselves with the task generally. Participants would often have trouble keeping their fixation within the gray box on the first few practice trials, where letters were presented peripherally above and/or below the box, but learned how to better navigate the task by the end of practice trials.

#### Experimental trials

Participants completed 112 experimental trials—14 blocks of 8 trials. Again, each block began with a diagram previewing the upcoming remapping. Each of the six remapping conditions and the control condition was completed in a random order in the first seven blocks and repeated in reverse order in the final seven blocks.

### Data processing

Data from 39 participants were entered into analyses. Each time the participant pressed the spacebar to advance to the next portion of the sentence during a trial, timestamps were collected and used to calculate reading speeds and to parse eye-tracking data.

#### Reading speed

Reading speed was calculated using a standardized word length of six characters (Carver, 1976; Mansfield et al., 1993; Mansfield et al., 2019) and the amount of time spent reading the entire sentence across each of the three screens. Reading speed was penalized for errors (see “Trial structure”), each of which was entered into the reading speed calculation as one fewer standardized word read.

#### Eye tracking

A custom script processed the raw eye-tracking data on each trial. A velocity threshold of 30°/second identified saccades, a loss of pupil data identified blinks, and fixations were detected using a moving window approach to identify fixations of at least 90 ms and a dispersion of less than 0.75° (see Salvucci & Goldberg, 2000). This script was used to parse the raw eye-tracking data into saccade, fixation, and blink events, which were then analyzed for spatial position and duration (see Supplementary Materials for additional eye-tracking results).

#### Analysis

Standard-length word reading speed was first analyzed for each participant for each of the six remapping strategies as a percentage of the control condition to probe the best-performing remapping. This analysis and accompanying fourfold validation were conducted using custom scripts in MATLAB. Subsequent analyses used linear mixed models (LMMs) using R (4.5.1) with lme4 (Bates, Mächler, Bolker, & Walker, 2015) and lmertest (Kuznetsova, Brockhoff, & Christensen, 2017) packages. For each analysis, four models were compared with all combinations of the fixed factors of scotoma shape, remapping strategy, and interaction, along with the random factor of participant:

M1: DV ∼ ScotomaShape + RemappingStrategy + ScotomaShape * RemappingStrategy + (1 | Participant)
M2: DV ∼ ScotomaShape + RemappingStrategy + (1 | Participant)
M3: DV ∼ RemappingStrategy + (1 | Participant)
M4: DV ∼ ScotomaShape + (1 | Participant)

The model that produced the lowest Akaike information criterion (AIC; Sakamoto, Ishiguro, & Kitagawa, 1988) was analyzed. Pairwise comparisons were conducted on the estimated marginal means of these models using the emmeans package (Lenth, 2025), and effect sizes were calculated using the effectsize package (Ben-Shachar, Lüdecke, & Makowski, 2020). Descriptive condition means averaged first within and then across participants are reported within the text and result graphs, with error bars representing the standard error of the mean. Solid black horizontal lines on the result graphs represent performance in the control condition without a simulated scotoma or remapping for reference.

## Results

We investigated how the different remapping strategies affected reading speed and whether their impact varied with the shape of the simulated scotoma. Our first hypothesis was that reading would be faster with remapping than without remapping when a simulated scotoma was present. To test this, we identified the best remapping for each participant and compared it to their reading speed in the no-remapping condition. Surprisingly, even though some remapping strategies elicited slow reading speeds across participants (see below), every remapping led to the fastest reading speed for at least one participant, and the no-remapping condition never led to the fastest reading speed (see Table 1). The remapping that resulted in the fastest reading speed also varied with the shape of the scotoma simulated. Of particular note, the horizontal gap remapping led to the fastest reading speed for all participants with the vertically elongated scotoma. For the other simulated scotomata, different remapping strategies resulted in the fastest reading speed.

**Table 1.** Frequency of how many participants read the fastest out of all the remappings for each remapping.

	Scotoma shape			
Strategy	Circle	Horizontal	Vertical	Total
Max row	0	1	0	1
Diagonals	4	2	0	6
Vertical gap	3	8	0	11
Horizontal gap	4	1	13	18
Max accuracy	2	1	0	3
No remap	0	0	0	0

**Figure 5.**
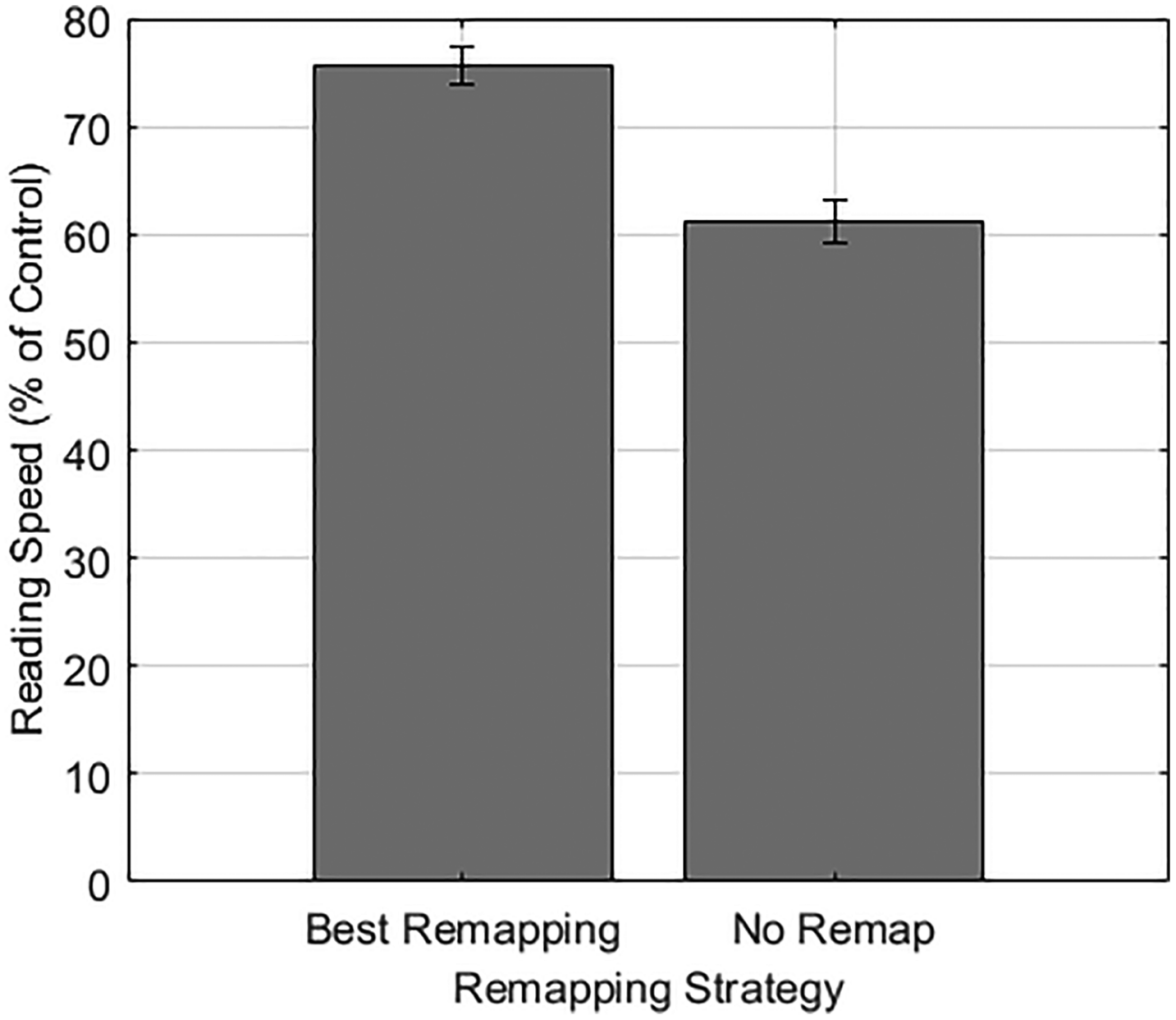
Reading speed results as a percentage of control reading speed for best remapping and no-remapping conditions.

We also quantified the effect of remapping on reading speed. Reading speeds were normalized to a percentage of control reading speed without a simulated scotoma or remapping. A paired *t*-test revealed that the best remapping for each participant (75.64% of control reading speed) resulted in faster reading than no remapping (61.10% of control reading speed), *t*(38) = 12.83, *p* < 0.01, *d* = 2.05 (see Figure 5). This difference equates to a 23.80% improvement in remapping compared to no remapping. To control for chance determining the best remapping, this *t*-test was confirmed with a fourfold cross-validation. Three-fourths of the data were used to identify the best remapping, and that remapping was compared to the no-remapping condition in the remaining quarter of the data. This was repeated for each quarter of the data as the “left-out” condition, and all confirmed the finding (*p*s < 0.01).

**Figure 6.**
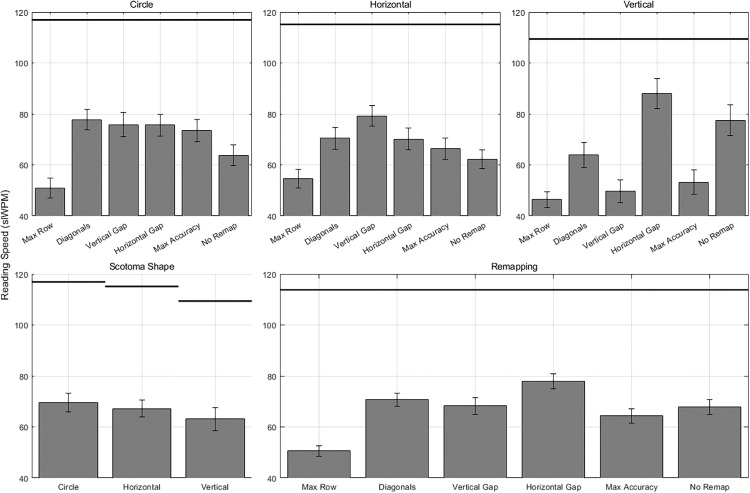
Reading speed results in standard-length words read per minute. (Top) Mean reading speed for each remapping strategy for each scotoma shape. (Bottom Left) Mean reading speed for each scotoma shape averaged over all remapping conditions. (Bottom Right) Mean reading speed for each remapping strategy averaged over all scotoma shapes. The black line above the bars represents reading speed for the control condition without a simulated scotoma or remapping.

Our second hypothesis was that customization of remapping based on the shape of the scotoma is important. That is, different remapping strategies should lead to faster reading in the presence of scotomata of different shapes. To test this, we analyzed reading speeds with the six different remapping strategies across and within each of the scotoma shape conditions. Reading speed was best predicted by the omnibus LMM containing the fixed effects of scotoma shape, remapping strategy, and the interaction, along with the random effect of participant, as confirmed by the lowest value AIC. The significant interaction, *F*(10, 3,620) = 63.88, *p* < 0.01, $\eta_{p}^{2}$ = 0.15, indicated that the effectiveness of the remapping strategies differed based on the shape of the simulated scotoma (see Figure 6, top), supporting the hypothesis.

**Figure 7.**
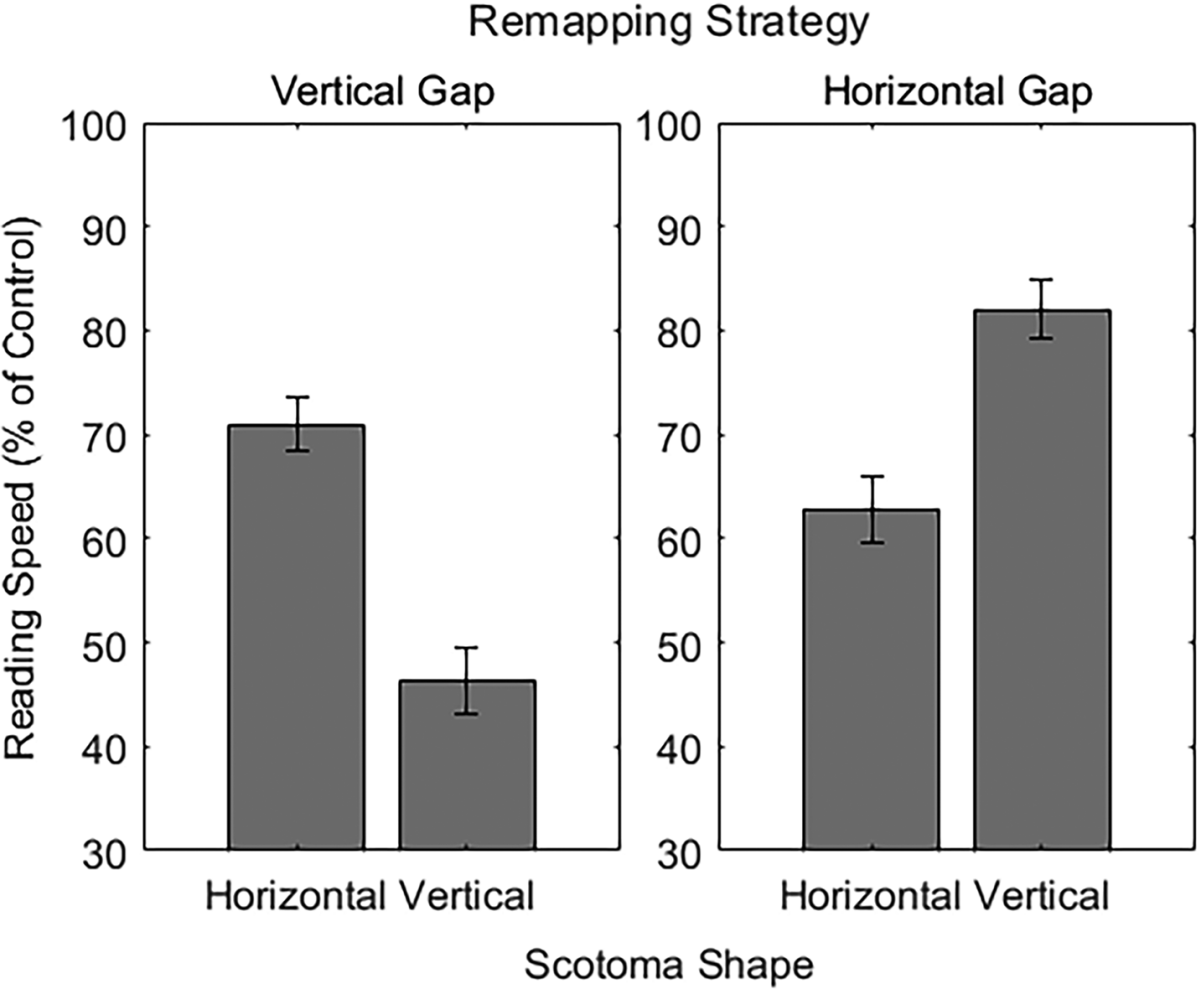
Reading speed results as a percentage of control reading speed for the horizontal gap and vertical gap remapping strategies and the horizontal and vertical scotomata.

Before reporting the other effects in the omnibus LMM, we report further probes of the interaction between scotoma shape and remapping. We examined reading speed only for the horizontally and vertically elongated scotomata for the horizontal and vertical gap remapping strategies using the same structured LMM. These conditions were chosen based on a strong a priori hypothesis from prior work (Flowers et al., 2024): We expected that the horizontal gap remapping would lead to faster reading with the vertically elongated scotoma than with the horizontally elongated scotoma because the letters need to be shifted further into the horizontal periphery for the horizontally elongated scotoma. We expected the opposite for the vertical gap remapping, where letters were shifted further into the vertical periphery for the vertically elongated scotoma. This hypothesis was confirmed by a significant interaction: The vertical gap remapping did lead to faster reading for the horizontal scotoma, and the horizontal gap remapping led to faster reading for the vertical scotoma, *F*(1, 782.05) = 350.58, *p* < 0.01, $\eta_{p}^{2}$ = 0.31 (see Figure 7).

Beyond the interactions between scotoma shape and remapping strategies, the omnibus LMM revealed that the different remapping strategies predicted reading speed, *F*(5, 3,620) = 150.20, *p* < 0.01, $\eta_{p}^{2}$ = 0.17 (see Figure 6, bottom right). Of note, the horizontal gap remapping resulted in faster reading overall than all other remapping conditions, including no remapping (post hoc comparisons, all *p*_Holm_ < 0.05). This pattern could result from the fact that horizontal gap remapping is the only remapping condition that presents all the letters along the horizontal midline, which may be easier to read. The three scotoma shapes did not differ significantly in how strongly they affected reading overall, *F*(2, 36) = 0.69, *p* > 0.05 (see Figure 6, bottom left), which was not surprising given that they were all roughly the same size.

### Fixation location

The six remapping strategies differed in whether and how remapped text was placed above or below the horizontal midline, and this may have affected how easily participants maintained their gaze within the gray box required for presentation of text. (We assume that fixating within the box and having the letters presented on the screen is an optimal strategy for reading.) To assess differences in maintaining gaze within the gray box, we analyzed the vertical position of fixations relative to the gray box (inside or outside).

**Figure 8.**
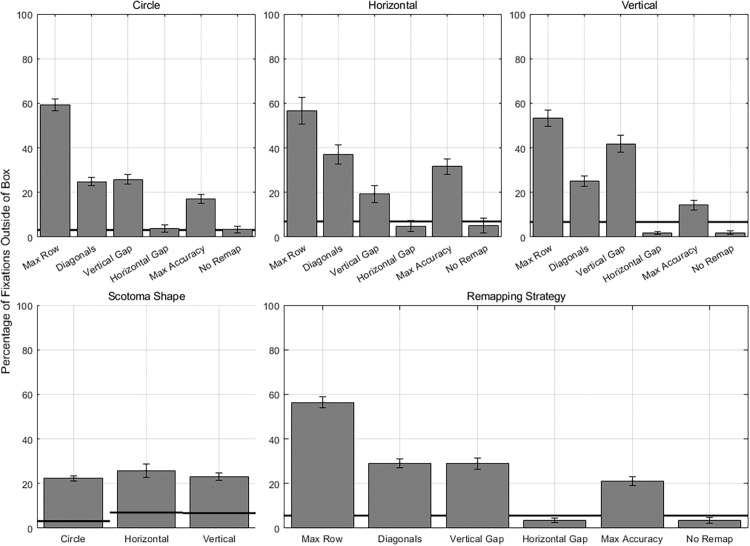
Percentage of fixations outside the box. (Top) Mean percentage of fixations outside the box for each remapping for each scotoma shape. (Bottom Left) Mean percentage of fixations outside the box for each scotoma shape, averaged over remapping strategies. (Bottom Right) Mean percentage of fixations outside the box for each remapping, averaged over scotoma shape. Error bars represent the standard error of the mean.

Again, the omnibus LMM yielded the lowest AIC value and was analyzed. A significant effect of remapping was found on the percentage of fixations outside of the box, *F*(5, 3,620) = 1,612.38, *p* < 0.01, $\eta_{p}^{2}$ = 0.69 (see Figure 8). The lowest percentage of fixations outside the box was found for horizontal gap (3.55%) and no remapping (3.52%), which did not significantly differ from each other (*p*_Holm_ > 0.05) but were significantly lower than all other remapping strategies (all *p*_Holm_ < 0.01). Max accuracy had the next lowest percentage of fixations outside the box (21.08%; lower than all of the following remapping strategies [all *p*_Holm_ < 0.01]), followed by vertical gap (28.96%) and diagonals (29.00%), which were not significantly different from each other (*p*_Holm_ > 0.05). Finally, max row (56.37%) had the highest percentage of fixations outside the box (all *p*_Holm_ < 0.01). Max accuracy may have had a lower percentage because its letters fell both above and below the horizontal midline. There was no significant effect of scotoma shape, *F*(2, 35.9) = 0.62, *p* > 0.05. A significant interaction between remapping and scotoma shape was observed, *F*(10, 3,620) = 71.44, *p* < 0.01, $\eta_{p}^{2}$ = 0.16. This interaction can be explained by the higher percentage of fixations outside the box in the vertical gap condition for the vertically elongated scotoma, where letters were shifted into the far periphery.

## Discussion

We measured whether customized spatial remapping could aid sentence reading when participants could freely move their eyes and were viewing the text with differently shaped simulated scotomata. Our first hypothesis was that customized spatial remapping would benefit reading. Strong evidence for this hypothesis was obtained: For each of the 39 participants, at least one of the remapping strategies resulted in faster reading than the no-remapping condition with a simulated scotoma, with an average improvement of 23.80% (range: 1.5%–62.8%). Our second hypothesis, that different remapping strategies would be best for different scotoma shapes, was also strongly supported, stressing the importance of customizing remapping: Reading was faster in the vertically elongated scotoma for horizontal gap remapping than for vertical gap remapping; the opposite was true for the horizontally elongated scotoma. Vertical gaps shifted letters too far away with the vertically elongated scotoma, and horizontal gaps shifted letters too far away with the horizontally elongated scotoma, negatively impacting reading speed.

Our results extend the previous findings that tested the efficacy of remapping (Aguilar & Castet, 2017; Gupta et al., 2018). Gupta et al. (2018) used a circular scotoma and a single remapping condition—a “Gaussian bump”—similar to the diagonals remapping here but that produced spatial distortions of the letters. They found large reading speed benefits of over 30% for larger scotomata (8°–16° diameters, masking nine or more letters) but not for a smaller scotoma (4° diameter, masking five to nine letters). Here, the scotomata were also smaller, masking three to five letters and ranged in width from 3.4° to 5.6°. When comparing each participant's best remapping to no remapping, a significant reading speed increase of 23.6% was observed. Thus, customizable spatial remapping improved reading speed for relatively small simulated scotomata. It may be expected that larger improvements would arise from larger scotomata.

The specific remapping strategies used here were previously tested in a word recognition task; there, the effectiveness of remapping strategies depended on the different shapes of the simulated scotomata, but the horizontal gap remapping was the best in all conditions (Flowers et al., 2024). The horizontal gap remapping also performed well in the sentence task here but was not as dominant—it was best only for the vertically elongated scotoma. The vertical gap remapping with the horizontal scotoma led to the fastest reading speed in 8 of the 13 participants who read with the horizontally elongated scotoma. The circular scotoma was created to favor neither vertical nor horizontal shifts of text and saw a relatively even distribution of remapping efficacy. Of note, the max row remapping was particularly bad at aiding reading in the current study, indicating that vertically displaced peripheral presentation of text interferes with oculomotor control during reading.

The lack of a clear best-performing remapping for the circular and horizontally elongated scotomata may indicate that different remapping strategies are similar in their ability to aid reading under these conditions. Alternatively, the participants tested here may have slight differences in their letter recognition across the visual field compared to that of the participants previously tested whose letter recognition maps were used to create the remapping strategies. It may be that if their letter recognition maps were collected individually, the remapping strategies may have differed. In either case, the strong improvement in reading speed for each participant's best-performing remapping demonstrates the utility of identifying the best remapping strategy on an individual basis.

The different remapping strategies were created from previously collected letter recognition maps averaging over multiple typically sighted viewers, with the assumption that letter recognition follows roughly the same pattern for typically sighted observers (Flowers et al., 2024). Here, the letter recognition maps and simulated scotomata were rather simple. The scotomata have well-defined boundaries and are continuous, while both they and the letter recognition maps are centered on the anatomic fovea, which typically sighted participants use for fixation. However, this need not be the case. The letter recognition maps allow both mapping of CFL to identify letters to be remapped and peripheral areas of good letter recognition to identify where to remap letters, regardless of boundaries or continuity. It is also flexible to artifacts in CFL, where a scotoma may not have a clear boundary separating CFL from spared vision.

A central purpose of the current work is its potential to aid readers with CFL. The maps for CFL readers would differ; readers with CFL often employ a PRL—a nonfoveal area of retina with spared vision used for fixation (Crossland, Engel, & Legge, 2011; Cummings, Whittaker, Watson, & Budd, 1985) and reading (Fidanci, Flowers, Larson, Engel, & Legge, 2025). As the location used for fixation is not occluded by a scotoma and would not result in poor letter recognition, letters would be presented at the PRL when remapped, following our perimetry-based remapping algorithm (see Figures 1E, 1F; Flowers et al., 2024). The scotoma, where letters would be remapped away from, would usually appear adjacent to the PRL rather than at the center of the grid. The work reported here still tests free reading along remapping trajectories in peripheral vision, centered on a location used for fixation. These general aspects of the experiment would still hold in actual CFL readers, giving us a reasonable expectation that what we learn here would generalize to them. We are currently testing more directly for the benefits of remapping in sentence reading in people with CFL.

Important differences between a pathological scotoma and the simulations used here might influence the generalizability of the current results to actual CFL. In particular, people with scotomata often develop a PRL (Crossland et al., 2011; Tarita-Nistor, Sverdlichenko, & Mandelcorn, 2023)—a functioning area of the retina that is used as a pseudo-fovea for navigating the visual field. Fixation with a PRL may not be as stable as foveal vision in typically sighted participants. Hence, testing customizable spatial remapping with readers with scotomata from macular disease is an important next step.

Beyond the use of a PRL, readers with scotomata exhibit different eye movement patterns (Rubin & Feely, 2009; Verghese et al., 2021; Wang et al., 2023) and studies aimed at training eye movements have proved useful (Seiple et al., 2011). Readers with CFL also have scotomata of different shapes, sizes, and qualities (e.g., opaqueness, continuity) than the narrow range simulated here. Eye-tracking measures have varied with the size of real (Rubin & Feely, 2009) or simulated scotoma (Yu & Kwon, 2023), suggesting that the best remapping strategy could vary as well.

The presence of a real or simulated scotoma forces visual processing to the periphery. Typically sighted viewers show variability in performance across a range of tasks in peripheral vision (Greenwood, Szinte, Sayim, & Cavanagh, 2017). Our results are consistent with this variability, as different remapping strategies resulted in the fastest reading speeds for different viewers with the same-shaped simulated scotoma. While the current study is framed in the context of CFL at its core, it relies on and is a test of peripheral processing and reading ability. The findings here may, therefore, be of interest to researchers interested in peripheral processing and reading capabilities beyond its applications to aiding CFL. Indeed, the importance of the functional periphery supports the motive of assessing peripheral capabilities beyond merely demarcating the bounds of the scotoma.

The method of spatial remapping employed here was limited by the use of a fixed grid, in a screen coordinate frame, of letter locations: Each letter in the remapped text was presented at the center of a box in the grid, and the remapping was computed relative to the center of the box nearest fixation. One advantage of this method was that it resulted in a finite number of possible displays, which could be precomputed and displayed quickly. It also had the benefit of reducing perceptual jitter in the displays, as small changes in fixation within a single grid location did not yield a change in what was presented. However, in this system, letters jumped from one box to another as participants moved their eyes; removing the grid constraint is an interesting topic for future research. It would allow letters to shift more continuously during reading, while requiring rapid on-the-fly computation and additional modifications to avoid adding jitter.

The current study included only a few practice trials. It is possible that the remapping strategies could alter reading speed differently with more training. In particular, remapping strategies with vertical displacement may negatively affect oculomotor control, as shown by the higher percentage of fixations away from the line of text in these conditions, and this could be improved with more training. Nevertheless, the observed differences support the principle that the different remapping strategies have a differential immediate impact on reading performance. Training can also allow typically sighted viewers to develop a PRL (Chen et al., 2019; Kwon, Nandy, & Tjan, 2013; Maniglia, Soler, & Trotter, 2020; Maniglia, Visscher, & Seitz, 2023); while participants in the current study did not do so, they may perform differently with a trained PRL, and the potential effects of that are an open question.

Participants are, therefore, currently fixating with their fovea, which is occluded by the simulated scotoma. This capitalizes on fixational control in typical vision that may be less reliable with field loss. Conversely, readers with CFL may better optimize peripheral viewing, especially at and near their PRL. Training can improve the use of a PRL (Chung, 2011; Yu, Legge, Park, Gage, & Chung, 2010) and may also have oculomotor control benefits such as suppressing unhelpful vertical eye movements. Many of the remapping strategies with vertical displacement of text led to a higher percentage of fixations outside of the box; perhaps training could lead to more improvement with these remapping strategies. Exploring the effects of practice with the different remapping strategies is a worthwhile area of future research.

## Conclusions

Spatial remapping improved reading speed with simulated scotomata, and the remapping strategy that led to the fastest reading depended on the shape of the simulated scotoma. Eye movement measures varied along with reading speed differences. Spatial remapping is a promising possibility for reading in viewers with CFL, where text can be shifted away from the scotoma and toward the PRL, and future work should explore this possibility in this population directly.

## Supplementary Material

Supplement 1

## References

[bib1] Aguilar, C., & Castet, E. (2017). Evaluation of a gaze-controlled vision enhancement system for reading in visually impaired people. *PLoS One,* 12, e0174910, 10.1371/journal.pone.0174910. 28380004 PMC5381883

[bib2] Bates, D., Mächler, M., Bolker, B., & Walker, S. (2015). Fitting linear mixed-effects models using lme4. *Journal of Statistical Software,* 67, 1–48, 10.18637/jss.v067.i01.

[bib3] Ben-Shachar, M. S., Lüdecke, D., & Makowski, D. (2020). Effect size: Estimation of effect size indices and standardized parameters. *Journal of Open Source Software,* 5, 2815, 10.21105/joss.02815.

[bib4] Brainard, D. H. (1997). The psychophysics toolbox. *Spatial Vision,* 10, 433–436. 9176952

[bib5] Bullimore, M. A., & Bailey, I. L. (1995). Reading and eye movements in age-related maculopathy. *Optometry and Vision Science,* 72, 125–138. 7753526 10.1097/00006324-199502000-00011

[bib6] Calabrèse, A., Owsley, C., McGwin, G., & Legge, G. E. (2016). Development of a reading accessibility index using the MNREAD acuity chart. *JAMA Ophthalmology,* 134, 398–405, 10.1001/jamaophthalmol.2015.6097. 26868760 PMC5369600

[bib7] Calabrese, A., To, L., He, Y., Berkholtz, E., Rafian, P., & Legge, G. E. (2018). Comparing performance on the MNREAD iPad application with the MNREAD acuity chart. *Journal of Vision,* 18, 8, 10.1167/18.1.8. PMC5774869 29351351

[bib8] Carver, R. P. (1976). Word length, prose difficulty, and reading rate. *Journal of Reading Behavior,* 8, 193–203, 10.1080/10862967609547176.

[bib9] Cheong, A. M., Lovie-Kitchin, J. E., Bowers, A. R., & Brown, B. (2005). Short-term in-office practice improves reading performance with stand magnifiers for people with AMD. *Optometry and Vision Science,* 82, 114–127, 10.1097/01.OPX.0000153244.93582.FF. 15711458

[bib10] Chen, N., Shin, K., Millin, R., Song, Y., Kwon, M., & Tjan, B. S. (2019). Cortical reorganization of peripheral vision induced by simulated central vision loss. *Journal of Neuroscience,* 39, 3529–3536, 10.1523/JNEUROSCI.2126-18.2019. 30814310 PMC6495137

[bib11] Chung, S. T. (2011). Improving reading speed for people with central vision loss through perceptual learning. *Investigative Ophthalmology & Visual Science,* 52, 1164–1170, 10.1167/iovs.10-6034. 21087972 PMC3053100

[bib12] Crossland, M. D., Engel, S. A., & Legge, G. E. (2011). The preferred retinal locus in macular disease: toward a consensus definition. *Retina,* 31, 2109–2114, 10.1097/IAE.0b013e31820d3fba. 21555970

[bib13] Cummings, R. W., Whittaker, S. G., Watson, G. R., & Budd, J. M. (1985). Scanning characters and reading with a central scotoma. *Optometry and Vision Science,* 62, 833–843. 10.1097/00006324-198512000-00004 4083327

[bib14] Fidanci, A., Flowers, C. S., Larson, C., Engel, S. A., & Legge, G. E. (2025). The preferred retinal locus for reading in central vision loss. *Investigative Ophthalmology and Vision Science,* 66(14), 12. 10.1167/iovs.66.14.12 PMC12599517 41196131

[bib15] Flowers, C. S., Legge, G. E., & Engel, S. A. (2024). Customizing spatial remapping of letters to aid reading in the presence of a simulated central field loss. *Journal of Vision,* 24, 1–19, 10.1167/jov.24.4.17. PMC11033602 38635281

[bib16] Gill, K., Mao, A., Powell, A. M., & Sheidow, T. (2013). Digital reader vs print media: The role of digital technology in reading accuracy in age-related macular degeneration. *Eye,* 27, 639–643, 10.1038/eye.2013.14. 23492860 PMC3650266

[bib17] Greenwood, J. A., Szinte, M., Sayim, B., & Cavanagh, P. (2017). Variations in crowding, saccadic precision, and spatial localization reveal the shared topology of spatial vision. *Proceedings of the National Academy of Sciences,* 114, E3573–E3582, https://doi.org/10.1073/pnas.1615504114. 10.1073/pnas.1615504114 PMC5410794 28396415

[bib18] Gupta, A., Mesik, J., Engel, S. A., Smith, R., Schatza, M., Calabrese, A., ... Legge, G. E. (2018). Beneficial effects of spatial remapping for reading with simulated central field loss. *Investigative Ophthalmology & Visual Science,* 59, 1105–1112, 10.1167/iovs.16-21404. 29490347 PMC5830989

[bib19] Hassell, J. B., Lamoureux, E. L., & Keeffe, J. E. (2006). Impact of age related macular degeneration on quality of life. *British Journal of Ophthalmology,* 90, 593–596. 16622089 10.1136/bjo.2005.086595 PMC1857044

[bib20] Ho, J. S., Loshin, D. S., Barton, R. S., & Juday, R. D. (1995). Testing of remapping for reading enhancement for patients with central visual field losses. In Huck F. O., & Juday R.D., eds. *Visual Information Processing IV* (Vol. 2488, pp. 417–424). Bellingham, WA: SPIE, 10.1117/12.211991.

[bib21] Kleiner, M., Brainard, D., Pelli, D., Ingling, A., Murray, R., & Broussard, C. (2007). What's new in Psychtoolbox-3. *Perception,* 36, 1–16.

[bib22] Kuznetsova, A., Brockhoff, P. B., & Christensen, R. H. B. (2017). lmerTest Package: Tests in linear mixed effects models. *Journal of Statistical Software,* 82, 1–26, 10.18637/jss.v082.i13.

[bib23] Kwon, M., Nandy, A. S., & Tjan, B. S. (2013). Rapid and persistent adaptability of human oculomotor control in response to simulated central vision loss. *Current Biology,* 23, 1663–1669, 10.1016/j.cub.2013.06.056. 23954427 PMC3773263

[bib24] Lenth, R. (2025). emmeans: Estimated marginal means, aka least-squares means. R package version 1.11.1-00001, https://rvlenth.github.io/emmeans/.

[bib25] Loshin, D. S., & Juday, R. D. (1989). The programmable remapper: Clinical applications for patients with field defects. *Optometry and Vision Science,* 66, 389–395. 2475839 10.1097/00006324-198906000-00009

[bib26] Maniglia, M., Soler, V., & Trotter, Y. (2020). Combining fixation and lateral masking training enhances perceptual learning effects in patients with macular degeneration. *Journal of Vision,* 20, 19, 10.1167/jov.20.10.19. PMC7571291 33064123

[bib27] Maniglia, M., Visscher, K. M., & Seitz, A. R. (2023). Consistency of preferred retinal locus across tasks and participants trained with a simulated scotoma. *Vision Research,* 203, 108158, 10.1016/j.visres.2022.108158. 36527839 PMC9914520

[bib28] Mansfield, J. S., Ahn, S. J., Legge, G. E., & Luebker, A. (1993). A new reading-acuity chart for normal and low vision. *Optical Society of America Technical Digest* 3, 232–235, 10.1364/NAVS.1993.NSuD.3.

[bib29] Mansfield, J. S., Atilgan, N., Lewis, A. M., & Legge, G. E. (2019). Extending the MNREAD sentence corpus: Computer-generated sentences for measuring visual performance in reading. *Vision Research,* 158, 11–18, 10.1016/j.visres.2019.01.010. 30731097 PMC6538455

[bib30] Massof, R. W., Rickman, D. L., & Lalle, P. A. (1994). Low vision enhancement system. *Johns Hopkins APL Technical Digest,* 15, 120–125.

[bib31] Murro, V., Sodi, A., Giacomelli, G., Mucciolo, D. P., Pennino, M., Virgili, G., ... Rizzo, S. (2017). Reading ability and quality of life in Stargardt disease. *European Journal of Ophthalmology,* 27, 740–745, 10.5301/ejo.5000972. 28430335

[bib32] Nguyen, N. X., Stockum, A., Hahn, G. A., & Trauzettel-Klosinski, S. (2011). Training to improve reading speed in patients with juvenile macular dystrophy: A randomized study comparing two training methods. *Acta Ophthalmologica,* 89, e82–e88, 10.1111/j.1755-3768.2010.02081.x. 21272283

[bib33] Nilsson, U. L., Frennesson, C., & Nilsson, S. E. G. (2003). Patients with AMD and a large absolute central scotoma can be trained successfully to use eccentric viewing, as demonstrated in a scanning laser ophthalmoscope. *Vision Research,* 43, 1777–1787, 10.1016/S0042-6989(03)00219-0. 12818347

[bib34] Rayner, K. (1978). Eye movements in reading and information processing. *Psychological Bulletin,* 85, 618, 10.1037/0033-2909.85.3.618. 353867

[bib35] Rayner, K. (1998). Eye movements in reading and information processing: 20 years of research. *Psychological Bulletin,* 124, 372, 10.1037/0033-2909.124.3.372. 9849112

[bib36] Rayner, K., Smith, T. J., Malcolm, G. L., & Henderson, J. M. (2009). Eye movements and visual encoding during scene perception. *Psychological Science,* 20, 6–10, 10.1111/j.1467-9280.2008.02243.x. 19037907 PMC2667830

[bib37] Rubin, G. S., & Feely, M. (2009). The role of eye movements during reading in patients with age-related macular degeneration (AMD). *Neuro-Ophthalmology,* 33, 120–126, 10.1080/01658100902998732.

[bib38] Sakamoto, Y., Ishiguro, M., & Kitagawa, G. (1988). Akaike information criterion statistics. *Journal of the Royal Statistical Society Series A: Statistics in Society*, 151(3), 567–568.

[bib39] Salvucci, D. D., & Goldberg, J. H. (2000). Identifying fixations and saccades in eye-tracking protocols. In *Proceedings of the 2000 Symposium on Eye Tracking Research & Applications*. New York: ACM Press; (pp. 71–78), 10.1145/355017.355028.

[bib40] Seiple, W., Grant, P., & Szlyk, J. P. (2011). Reading rehabilitation of individuals with AMD: Relative effectiveness of training approaches. *Investigative Ophthalmology & Visual Science,* 52, 2938–2944, 10.1167/iovs.10-6137. 21296824

[bib41] Seiple, W., Szlyk, J. P., McMahon, T., Pulido, J., & Fishman, G. A. (2005). Eye-movement training for reading in patients with age-related macular degeneration. *Investigative Ophthalmology & Visual Science,* 46, 2886–2896, 10.1167/iovs.04-1296. 16043863

[bib42] Tarita-Nistor, L., Sverdlichenko, I., & Mandelcorn, M. S. (2023). What is a preferred retinal locus? *Annual Review of Vision Science,* 9, 201–220, 10.1146/annurev-vision-111022-123909. 36944313

[bib43] Verghese, P., Vullings, C., & Shanidze, N. (2021). Eye movements in macular degeneration. *Annual Review of Vision Science,* 7, 773–791, 10.1146/annurev-vision-100119-125555. PMC8916065 34038144

[bib44] Wang, R., Zeng, L., Zhang, X., Mondal, S., & Zhao, Y. (2023). Understanding how low vision people read using eye tracking. In *Proceedings of the 2023 CHI Conference on Human Factors in Computing Systems* (pp. 1–17). New York: ACM, 10.1145/3544548.358123.

[bib45] Wensveen, J. M., Bedell, H. E., & Loshin, D. S. (1995). Reading rates with artificial central scotomata with and without spatial remapping of print. *Optometry and Vision Science,* 72, 100–114. 7753524 10.1097/00006324-199502000-00009

[bib46] Yu, D., Legge, G. E., Park, H., Gage, E., & Chung, S. T. (2010). Development of a training protocol to improve reading performance in peripheral vision. *Vision Research,* 50, 36–45, 10.1016/j.visres.2009.10.005. 19819251 PMC2794940

[bib47] Yu, H., & Kwon, M. (2023). Altered eye movements during reading with simulated central and peripheral visual field defects. *Investigative Ophthalmology & Visual Science,* 64, 21, 10.1167/iovs.64.13.21. PMC10584020 37843494

