# Supplementary material for: Spatial remapping improves reading with simulated central field loss
Source: J Vis. 2026 Apr 17;26(4):11. doi: 10.1167/jov.26.4.11 (PMC13101835; doi:10.1167/jov.26.4.11)
Supplement: Supplement 1 [file jovi-26-4-11_s001.docx]

**Supplementary Materials**

**Fixation Duration.** The omnibus LMM yielded the lowest AIC and was analyzed. A significant main effect of remapping was found, F(5, 3620) = 395.38, *p* < 0.01, $\eta_{p}^{2}$ = 0.35 (see Figure SM1 bottom right). Post hoc comparisons revealed that fixation duration was shortest for the horizontal gap followed by no remapping, which were both faster than all other conditions. Diagonals, max accuracy, and vertical gap were the next three fastest: fixation duration with the diagonals remapping was significantly shorter than with vertical gap, but neither significantly differed from max accuracy. Finally, max row was significantly longer than all others (all significant post hoc comparisons *p*_holm_ < 0.05). A significant effect of scotoma shape was not found, F(2,36) = 0.47, *p* > 0.05 (see Figure SM1 bottom left). A significant interaction between remapping and scotoma shape was found, F(10, 3620) = 27.77, *p* < 0.01, $\eta_{p}^{2}$ = 0.07 (see Figure SM1 top), which followed the pattern of the interaction in the reading speed data. It can be characterized by a larger difference in fixation duration in the vertically-elongated scotoma, whereby the horizontal gap exhibits a shorter fixation duration than the vertical gap remapping than in the other two scotoma shapes.


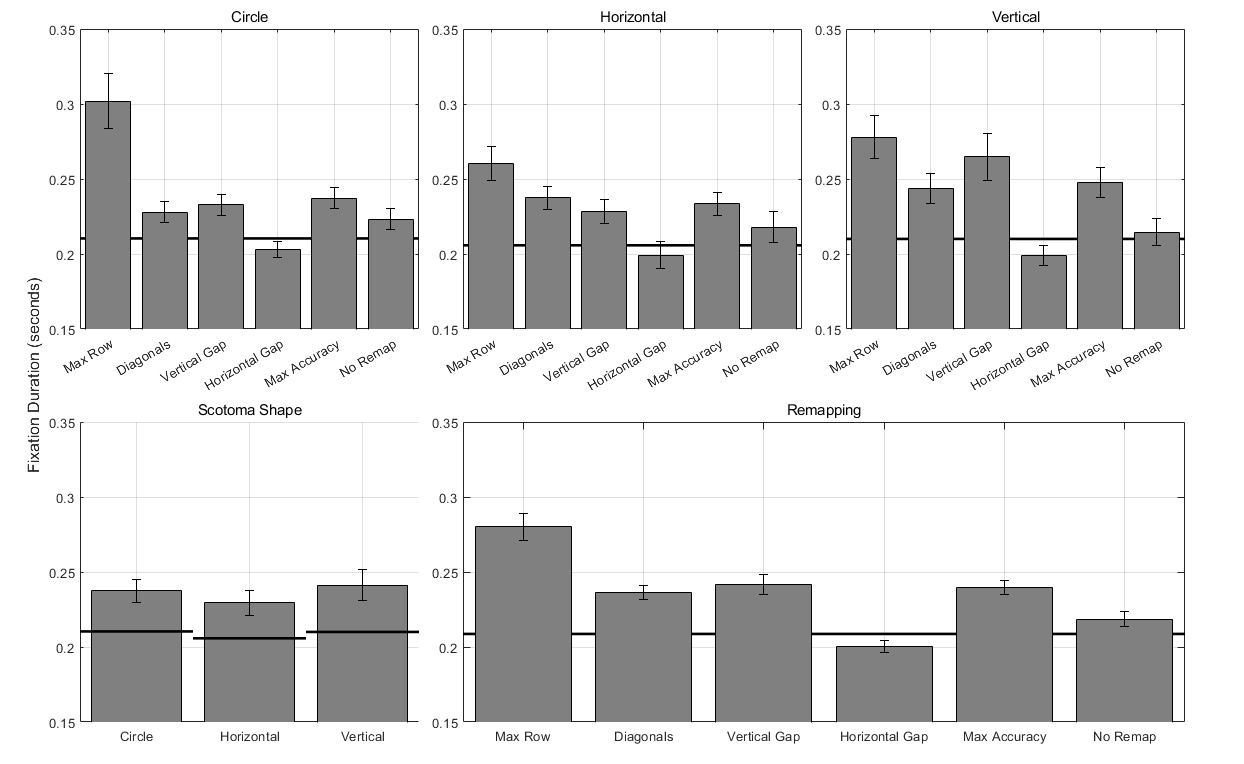


Figure SM1. Fixation duration results. Top) Mean fixation duration for each remapping for each scotoma shape. Bottom left) Mean fixation duration for each scotoma shape averaged over remapping strategies. Bottom right) Mean fixation duration for each remapping averaged over scotoma shape. Error bars represent standard error of the mean and solid black lines represent control performance.

**Saccade Amplitude.** The omnibus LMM yielded the lowest AIC and was analyzed. A significant effect of remapping was found, F(5, 3620) = 500.97, *p* < 0.01, $\eta_{p}^{2}$= 0.41 (see Figure SM2 bottom right). Post hoc comparisons revealed that horizontal gap remapping had the largest mean saccade amplitude followed by no remapping followed by vertical gap. Max accuracy and diagonals had the next largest saccade amplitudes and did not differ significantly from each other. Max row had significantly smaller saccade amplitudes than all other conditions (all significant post hoc comparisons *p*_holm_ < 0.05). There was not a significant effect of scotoma shape, F(2,36) = 0.23, *p* > 0.05 (see Figure SM2 bottom left). A significant interaction between remapping and scotoma shape was observed, F(10, 3620) = 21.55, *p* < 0.01, $\eta_{p}^{2}$= 0.06 (see Figure SM2 top). It is important to consider that the horizontal gap remapping strategy shifts letters left and right and occludes 1.5 (vertically-elongated scotoma) or 2.5 letters (circle, horizontally-elongated scotoma) in either direction, translating to 1.7° or 2.8° when interpreting the saccade amplitude results.


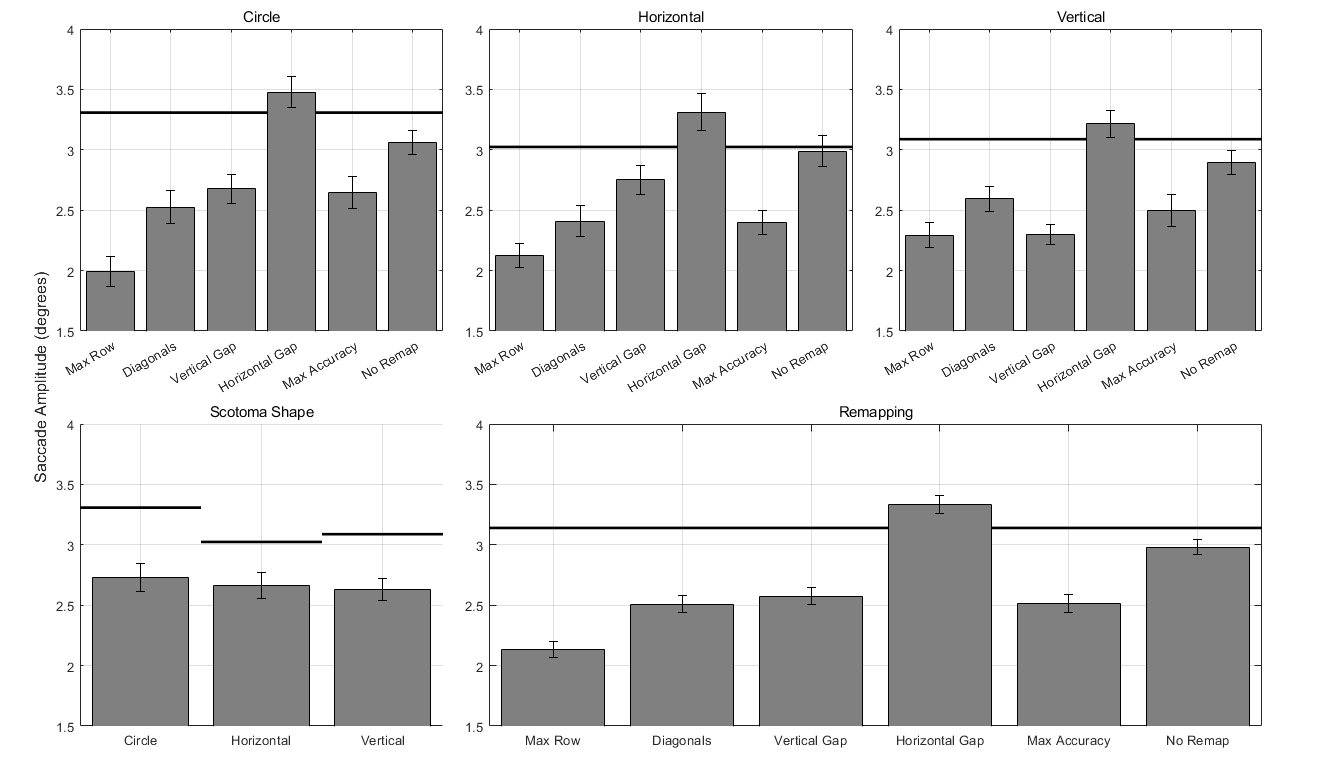


Figure SM2. Saccade amplitude results. Top) Mean saccade amplitude for each remapping for each scotoma shape. Bottom left) Mean saccade amplitude for each scotoma shape averaged over remapping strategies. Bottom right) Mean saccade amplitude for each remapping averaged over scotoma shape. Error bars represent standard error of the mean and solid black lines represent control performance.

**Regressive Fixations.** Starting with the second fixation on each screen, fixations after a forward fixation were categorized as either being a forward (to the right) or backward (to the left) fixation from the previous one. The rate of regressive fixations was the proportion of the detected backward fixations. The omnibus LMM yielded the lowest AIC and was analyzed. A significant main effect of remapping was found, F(5, 3620) = 11.00, *p* < 0.01, $\eta_{p}^{2}$= 0.01 (see Figure SM3 bottom right). No remapping resulted in a higher rate of regressive fixations than all other conditions. The only other significant differences were that max accuracy resulted in a higher rate of regressive fixations than the diagonals or vertical gap remapping. A significant effect of scotoma shape was not observed, F(2,36) = 0.54, *p* > 0.05 (see Figure SM3 bottom left). A significant interaction between scotoma shape and remapping was observed, F(5, 3620) = 16.77, *p* < 0.04, $\eta_{p}^{2}$ = 0.05 (see Figure SM3 top). This interaction was explained by a lower rate of regressive fixations in the horizontal gap remapping for the vertically-elongated scotoma.


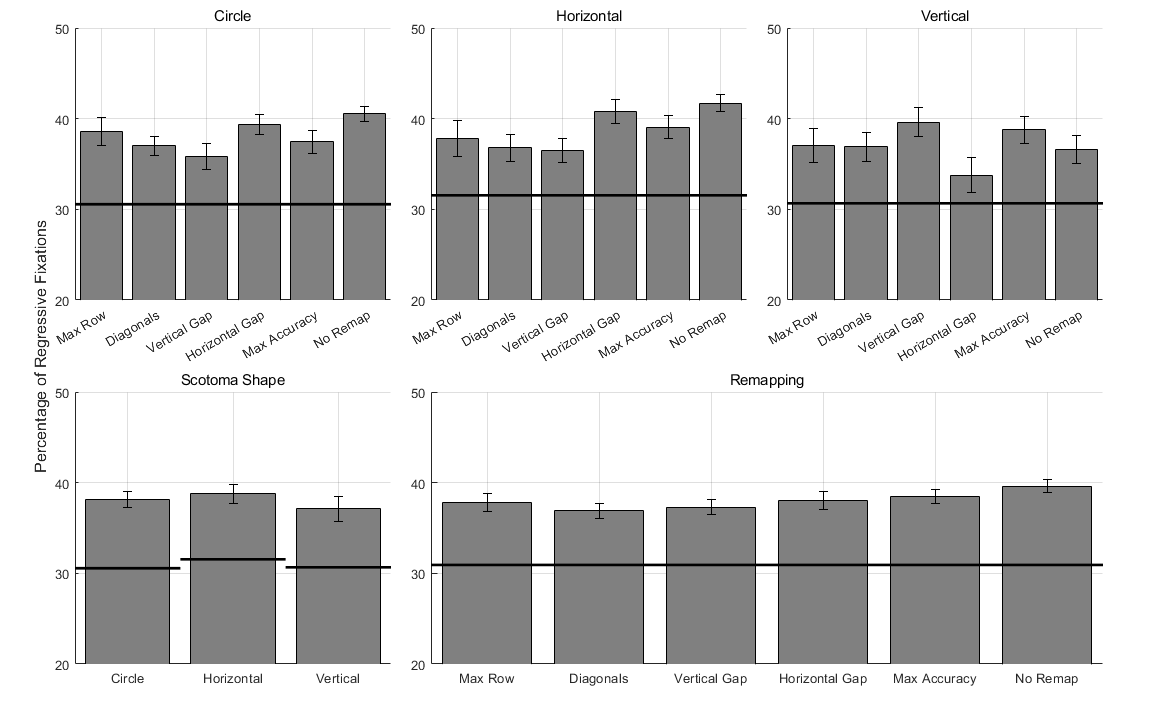


Figure SM3. Regressive fixations results. Top) Mean rate of regressive fixations for each remapping for each scotoma shape. Bottom Left) Mean fixation duration for each scotoma shape averaged over remapping strategies. Bottom Right) Mean fixation duration for each remapping averaged over scotoma shape. Error bars represent standard error of the mean and solid black lines represent control performance.
